# Improved chromatographic separation of modified deoxycytidine nucleosides enhances LC-MS sensitivity

**DOI:** 10.17912/micropub.biology.002202

**Published:** 2026-07-13

**Authors:** Christine Isaguirre, Molly T. Soper-Hopper, Stacey L. Thomas, Rachel Shereda, Darrell Chandler, Peter A. Jones, Ryan D. Sheldon

**Affiliations:** 1 Mass Spectrometry Core, Van Andel Institute, Grand Rapids, MI, United States; 2 Department of Epigenetics, Van Andel Institute, Grand Rapids, MI, United States

## Abstract

Cytosine methylation is an epigenetic modification that regulates transcription. DNA demethylation occurs through oxidation of methylcytosine to hydroxymethylcytosine, making hydroxymethylation an important and translationally relevant marker of DNA methylation dynamics. These epigenetic modifications can be measured in their deoxynucleoside forms from enzymatically digested DNA by liquid chromatography-mass spectrometry (LC-MS). However, 5-hydroxymethyl-2′-deoxycytidine (5hmdC) co-elutes with 2′-deoxycytidine (dC). Because 5hmdC represents <0.05% of total dC in DNA, co-elution with dC causes ion suppression that limits detection and quantitation. Here, we present a chromatographic method that separates 5hmdC from dC, eliminates ion suppression, and improves the sensitivity of 5hmdC detection.

**Figure 1. A mobile phase containing ammonium hydroxide separates 5hmdC from dC, alleviates ion suppression, and improves LC-MS detection sensitivity:** A) Reversed-phase chromatography does not separate 5hmdC from dC if the mobile phase contains 0.1% formic acid (acidic; top), but does separate the analytes when using 0.01% ammonium hydroxide (NH _4_ OH) and 1 mM ammonium acetate as the mobile phase (basic, bottom). (B) Increasing concentrations of dC suppress 5hmdC signal when the two analytes co-elute, but not when the analytes are separated with the basic mobile phase. Data are the mean ± s.d. from n=3 replicates, and were derived from a constant input of 50 ng mL ^-1^ 5hmdC in an increasing background of dC. The grey-shaded region indicates dC concentrations typically observed from DNA digests. (C) Separation increases LC-MS signal intensity of neat 5hmdC (top) and dC (bottom) standards, likely through improved ionization efficiency. P-value between groups is <0.0001. (D) The complete method separates all DNA nucleosides and modified deoxycytidine nucleosides with an elution order of 2′-deoxycytidine (dC), 5-hydroxymethyl-2′-deoxycytidine (5hmdC), 2’-deoxyguanosine (dG), 5-methyl-2’-deoxycytidine (5mdC), 2′-deoxythymidine (dT), and 2′-deoxyadenosine (dA). (E) Including deuterated 5hmdC (5hmdC-d _3_ ) as an internal standard enables absolute quantitation of 5hmdC. Data are from n=6 independent standard curves, and are the mean ± s.d. of log(5hmdC/5hmdC-d _3_ ). The dotted line is the linear regression of the log-transformed means. (F) Vitamin C (VC) treatment increases 5hmdC levels in HCT116 cells, but 5-aza-2′-deoxycytidine (5-Aza-CdR; decitabine) does not. Data are the mean ±s.d. from n=2 replicates per group, expressed as a percentage of dG to account for differences in DNA loading. These data demonstrate that the method is suitable for detecting an expected biological phenotype. (G) Same as panel F, except quantifying 5mdC. 5-Aza-CdR treatment decreases 5mdC concentration in HCT116 cells, as expected. N=2 replicates per condition. Table: Transition list for LC-MS acquisition of DNA nucleosides and corresponding internal standards. * denotes transition used for quantitation. Each fragment ion is separated by a comma, with corresponding fragmentor and collision energy (CE) voltages listed in the same order as their cooresponding fragment ions.

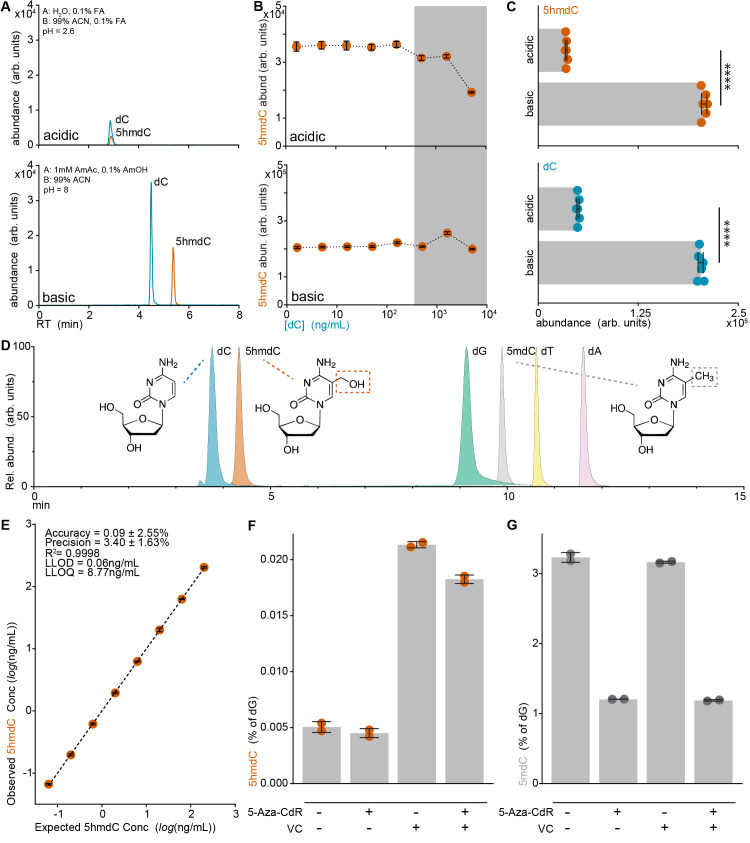



**Table:** 

Compound name	Precursor (m/z)	Product (m/z)	RT (min)	RT Window (min)	Fragmentor (V)	CE (V)	Polarity
2'deoxycytidine	228	112*, 95	4.3	2	50, 50	5, 40	+
2'deoxycytidine-13C515N	231	115*, 157, 98	4.3	2	55, 140, 50	30, 15, 40	+
5-hydroxymethyl-2'deoxycytidine	258	124*, 81	5.3	2	80, 80	30, 30	+
5-hydroxymethyl-2'deoxycytidine-d3	261	127*, 145	5.3	2	50, 90	30, 5	+
2'deoxyguanosine	268	152*, 135, 110	7.7	2	25, 40, 5	20, 50, 40	+
2'deoxyguanosine-13C515N	271	155*, 117	7.7	2	30, 100	5, 15	+
5-methyl-2'deoxycytidine	242	126*, 108	9.1	2	50, 50	10, 40	+
5-methyl-2'deoxycytidine-d3	245	129*, 117	9.1	2	50, 55	10, 15	+
2'deoxythymidine	243	127*, 117	9.2	2	50, 50	10, 10	+
2'deoxyadenosine	252	136*, 119	10.1	2	50, 100	10, 50	+

## Description

Naturally modified DNA bases and their associated DNA:protein interactions are fundamental to normal biology (Gommers-Ampt & Borst, 1995; Raiber et al., 2017). 5-Methylcytosine, in particular, is a key transcriptional regulator (Jones & Takai, 2001) with genome-wide DNA methylation patterns established in early embryonic development (Li, 2002). These patterns are maintained across cell divisions and are considered relatively stable across the lifespan (Bird, 2002). However, the discovery that TET enzymes can actively oxidize 5-methylcytosine to 5-hydroxymethylcytosine (Tahiliani et al., 2009) revealed that DNA methylation is more dynamic than originally thought (Wu & Zhang, 2010). Importantly, 5-hydroxymethylcytosine levels in tissues or circulating cell-free DNA are diagnostic hallmarks of various cancers and other diseases (Buscarlet et al., 2015; Chen et al., 2016; Li & Liu, 2011; Miao et al., 2015; Song et al., 2017; Song et al., 2025; Xu & Gao, 2020; Yu et al., 2021), with the distribution and ratio of 5-methylcytosine to 5-hydroxymethylcytosine providing important clues about the tissue-of-origin (Li et al., 2024; Li et al., 2017; Song et al., 2017), disease stage (Li et al., 2024; Liao et al., 2016), treatment response (Chen et al., 2021; Lian et al., 2012; Shao et al., 2024), and patient outcomes (Chen et al., 2017; Chiu et al., 2019; Dong et al., 2015; Liao et al., 2016; Orr et al., 2012). Accurate and sensitive quantitation of 5-hydroxymethylcytosine is therefore important for detecting epigenetic dysregulation and supporting biomarker development.

LC-MS is frequently used to quantify the deoxynucleosides of modified cytosine bases, including 5-hydroxymethyl-2′-deoxycytidine (5hmdC), 5-methyl-2′-deoxycytidine (5mdC), and unmodified 2’-deoxycytidine (dC) from enzymatically digested DNA. Most commonly, reversed phase separations are performed using mobile phases containing formic acid. In such methods, however, 5hmdC co-elutes with the far more abundant analyte dC, which limits sensitivity and quantitative accuracy of 5hmdC measurements ( Figure 1A, top). To address this, we evaluated several chromatography parameters, including stationary phase (BEH Amide, phenylhexyl), flow rate and gradient, and mobile phase salt addition (10 mM ammonium formate), and discovered that adding 0.01% ammonium hydroxide (NH _4_ OH) with 1 mM ammonium acetate (basic) to the mobile phase, instead of 0.1% formic acid (acidic), resulted in baseline chromatographic separation of dC and 5hmdC ( Figure 1A, bottom).

We next compared the amount of ion suppression observed with each solvent system by titrating 50 ng mL ^-1^ 5hmdC into half-log dilutions of dC (range = 5 µg mL ^-1^ to 1.58 ng mL ^-1^ ). Acidic chromatography decreased 5hmdC signal at increasing dC concentrations ( Figure 1B, top), indicating ion suppression of 5hmdC by dC. With the basic mobile phase, however, there was no detectable ion suppression ( Figure 1B, bottom). We also saw a marked increase in signal intensity for dC and 5hmdC with the basic method ( Figure 1C ), presumably from enhanced ionization efficiency and/or less in-source adduct/fragment formation. Thus, using 0.01% NH _4_ OH with 1mM ammonium acetate in the mobile phase alleviates ion suppression and improves signal intensity.

We then expanded the method to include 5-methyl-2′-deoxycytidine (5mdC) and the other DNA nucleosides, which cleanly separate over a 19-minute analytical gradient and are easily detected by MS ( Figure 1D ). Because dC, 5mdC, and 5hmdC are typically reported as a percentage of dG to account for DNA loading differences, we extended this method to include isotopically labeled internal standards of these four deoxynucleosides. Transitions for these internal standards are included in Table 1. For absolute quantitation, we prepared a stock mixture for each nucleoside at concentrations expected in 500 ng DNA, serially diluted the mix, and then separated the analytes with the basic method to generate a calibration curve. This approach enabled absolute quantitation of 5hmdC with 0.09 ± 2.6% accuracy and 3.4 ± 1.6% precision across six independently prepared standard curves ( Figure 1E ; transitions for all compounds and internal standards are included in Table 1; * denotes quantifier transitions). Lower limit of detection (LLOD) and lower limit of quantitation (LLOQ) were determined to be 0.06 ng mL ^-1^ and 8.77 ng mL ^-1^ , respectively.

Finally, as a proof of principle, we evaluated whether the method was sufficient to detect expected biological effects. HCT116 cells were treated with 5-aza-2′-deoxycytidine (5-Aza-CdR, decitabine) and/or vitamin C to decrease 5mdC and increase 5hmdC, respectively (Liu et al., 2016). DNA was isolated, digested to nucleosides, and used to determine absolute concentrations of 5mdC, 5hmdC, dC, and dG. As expected, vitamin C increased 5hmdC content independently of 5-Aza-CdR, whereas 5-Aza-CdR decreased 5mdC independently of vitamin C. These data demonstrate the utility of this method to detect biologically relevant epigenetic cytosine modifications.

## Methods

**Cell culture.** HCT116 cells were seeded on Day -1 at 300,000 cells per 100 mm dish in McCoy’s 5A medium (16600-082, Gibco) supplemented with 1% Pen Strep (15140-122, Gibco) and 10% FBS (F0926, Sigma-Aldrich). On Day 0, cells were treated with 300 nM 5-aza-2′-deoxycytidine (5-Aza-CdR) as a single dose. Culture medium was replaced on Day 2. Vitamin C was administered at 57 µM once daily for 5 consecutive days (Days 0-4). Cells were harvested on Day 5 for downstream analysis.

**DNA preparation** . DNA was purified and digested using an AllPrep DNA/RNA Mini Kit (80204, Qiagen). After following the kit protocol, DNA was eluted with 50 µL of LC/MS grade water (W6, Fisher) and further purified using the Genomic DNA Clean & Concentrator-25 (D4065, Zymo Research). DNA was again eluted with 50 µL of LC/MS grade water, and the DNA concentration of each sample was measured using a Qubit Fluorometer. The DNA was then degraded to single nucleosides using Nucleoside Digestion Mix (M0649S, NEB) by incubating 50 µL of sample containing 500-800 ng of DNA with 5 µL of enzyme mix for 4 hr at 37°C. The completeness of digestion was evaluated by running 10 µL of each sample on a 1.5% agarose gel, looking for the absence of ethidium bromide/SYBR Safe fluorescent signal. 100 ng of DNA was used as a positive control. When complete digestion was confirmed, the remaining DNA digest mixture was stored at -80°C until LC/MS sample preparation and analysis.

**Nucleoside preparation for LC-MS** . 35 µL of DNA digests and standard curve samples were extracted with 315 µL of 80% methanol (v/v) (Fisher, A456) containing [ ^13^ C _5_^15^ N]2’-deoxycytidine (100 ng/mL) (D239552, Toronto Research Chemicals), [D _3_ ]5-methyl-2’-deoxycytidine (200 ng/mL) (M295902, Toronto Research Chemicals), [D _3_ ]5-hydroxy-methyl-2’-deoxycytidine (200 ng/mL) (H946632, Toronto Research Chemicals), and [ ^13^ C _5_ ] 2’-deoxyguanosine (200 ng/mL) (D232617, Toronto Research Chemicals) as internal standards. After adding extraction solvent, samples were pulse vortexed, sonicated in a water bath for 5 min, and incubated on wet ice for one hr. Samples were centrifuged at 17,000 x g at 4°C for 10 min, and 280 µL of the supernatant was collected and dried overnight in a rotary vacuum evaporator. Samples were resuspended in LC/MS grade water, pulse vortexed, and sonicated in a water bath for 5 min. Samples were then transferred into autosampler vials (Fisher, 6PSV9-03FIVP) for LC-MS analysis.

**LC parameters** . 2 μL of each sample was injected and separated using a 21-minute gradient on a Cortecs T3 column (1.6 μm, 2.1 mm × 150 mm, 186008500, Waters, Eschborn, Germany) fitted with a Cortecs T3 VanGuard pre-column (1.6 μm, 2.1 mm × 5 mm, 186008508, Waters). Mobile phases for the acidic solvent system were A: LC/MS grade water and B: 99% acetonitrile (A955, Fisher), both with 0.1% (v/v) formic acid (A117, Fisher). For the basic solvent system, mobile phase A consisted of LC/MS grade water with 1 mM ammonium acetate (73594, Sigma) and 0.01% ammonium hydroxide. Mobile phase B consisted of 99% acetonitrile (A955-4, Fisher) and 1% LC/MS grade water. The column temperature was kept at 30°C, the flow rate was held at 0.3 mL min ^-1^ , and the chromatography gradient was as follows: 0-6 min held at 0% B; 6-9 min ramp from 0 to 10% B; 9-14 min ramp from 10% to 50% B; and 14-18.9 min ramp from 50% to 99% B. At 19 min, the mobile-phase composition was changed to 0% B and the flow rate was changed to 0.4 mL min ^-1^ and held until 20.5 min, then decreased to 0.3 mL min ^-1^ by 21 min.

**MS parameters** . Samples and standards were separated on an Agilent1290 Infinity II ultra-high performance liquid chromatography system analyzed with an Agilent 6470 triple quadrupole mass spectrometer. Mass spectrometer parameters were: gas flow at 13 L min ^-1^ at 80°C; sheath gas flow at 11 L min ^-1^ at 275 °C; and the nebulizer was set to 30 psi. Capillary voltage was +2500 V and nozzle voltage was +500 V. Data were acquired using dynamic multiple reaction monitoring (dMRM) with at least two transitions per compound. The transition list was developed and optimized using neat analytical standards, with parameters provided in Table 1. The dMRM parameters were determined based on running LC/MS grade analytical standards for each target compound. Peak picking and integration was performed using Skyline software (v 24.1).

**Absolute concentration** . A stock standard curve mix was prepared with individual analyte concentrations selected to account for their endogenous levels. The stock mix contained 2’-deoxycytidine (dC; 250 µg mL ^-1^ ; Caymen Chemical #30125), 5-methyl-2’-deoxycytidine (5mdC; 10 µg mL ^-1^ ; Caymen Chemical #16166), 5-hydroxy-methyl-2’-deoxycytidine (5hmdC; 0.5 µg mL ^-1^ ; Caymen Chemical #18162), and 2’-deoxyguanosine (dG; 250 µg mL ^-1^ ; Caymen Chemical #9002864). This stock was diluted 2.5X to generate the initial dilution, then serially diluted in half-log steps. Each dilution was then extracted as described above.

For each analyte, peak area was expressed as the ratio of analyte signal to the corresponding internal standard signal, and the response ratio was log-transformed. Expected analyte concentrations were also log-transformed, and linear regression of log-transformed response ratio versus log-transformed concentration was used to generate the calibration curve. For biological samples, analyte concentrations were interpolated from this calibration curve. The resulting concentrations (ng/mL) were then used to calculate the ratios 5hmdC/dG and 5mdC/dG, which were expressed as percentages to account for variation in DNA loading.

**Statistical analysis** . Standard curve concentrations and unknown response ratios (i.e. signal normalized to the cognate internal standard) were log-transformed, and linear regression was used to interpolate concentrations. Back-transformed concentrations were used to calculate accuracy as ( *observed-expected)/expected* x 100 and precision as ( *standard deviation)/mean* x 100 of observed versus expected concentrations.

The lower limit of detection and lower limit of quantitation were estimated from blank responses on the log scale. Blank responses were expressed as log _10_ (signal/internal standard signal), and the mean and standard deviation of the blank responses were calculated. The detection and quantitation thresholds on the response scale were defined as Ȳ _blank_ + 3.3s _blank_ and Ȳ _blank_ + 10s _blank_ , respectively. A calibration curve was generated by linear regression of log _10_ (response ratio) versus log _10_ (known concentration) according to Y = 0.9867X – 2.280. The corresponding log-transformed concentration was calculated as X = (Y + 2.280)/0.9867, and the final concentration was obtained by back-transformation as 10 ^X^ . All data are represented as means ± s.d.
